# Updating and refining estimates of typhoid fever burden for public health action

**DOI:** 10.1016/S2214-109X(14)70306-7

**Published:** 2014-10

**Authors:** John A Crump

**Affiliations:** Centre for International Health, University of Otago, PO Box 56, Dunedin 9054, New Zealand

Efforts to estimate the global burden of typhoid fever can be traced to a meeting of the Pan American Health Organization in 1984 and publication of the outcome in 1986.^1^ Although an important first step, the 1984 study was recognised as having a number of limitations including provision of scanty methodological detail, the availability of few source data, exclusion of China from the estimate, and lack of consideration of the age distribution of typhoid fever. Subsequently the global typhoid burden was re-estimated for the year 2000, accounting for growth of the global population, new typhoid fever incidence data from population-based studies and the control groups of vaccine trials, advances in the understanding of the age distribution of typhoid fever and its relation to force of infection, adjustment for blood culture sensitivity, and formalisation of methods for assessment of disease burden.^2^ Since 2000, an updated review of population-based studies of typhoid fever incidence and data from notifiable disease reports from countries with advanced surveillance systems has been published.^3^ Incorporating these data, the Institute for Health Metrics and Evaluation (IHME) added their first estimate of disability and death associated with typhoid and paratyphoid fevers in aggregate to the Global Burden of Disease (GBD) 2010 project.^4,5^ The IHME GBD 2010 estimate could be criticised for insufficient methodological detail for external reproducibility, lack of disaggregation of typhoid and paratyphoid fevers, little description of the age distribution of disease, and the surprising selection of liver abscesses and cysts as the prime disease complication of interest.^6^

It is in this context that Vittal Mogasale and others revisit typhoid fever burden with an eye to refining estimates to inform vaccine policy.^7^ Theirs is not a global estimate, although most typhoid fever cases do occur in countries classified in the low-income and middle-income group. Furthermore, with monovalent typhoid vaccines in mind, the focus is exclusively on *Salmonella enterica* serovar Typhi, with no estimate for *Salmonella* Paratyphi A or for invasive non-typhoidal *Salmonella*. The investigators did a series of well described systematic reviews to update and improve estimates of typhoid fever incidence, including age distribution, blood-culture sensitivity, and case-fatality ratio. They also take the innovative step of adding a risk-factor-based adjustment of typhoid fever incidence that accounts for lack of access to improved water in rural areas and in urban slums. This adjustment was derived from a further systematic review of case-control studies to ascertain the contribution of waterborne transmission to typhoid fever risk. In so doing, Mogasale and colleagues estimate that 11·9 million typhoid fever illnesses and 129 000 deaths occurred in low-income and middle-income countries 2010. These numbers are lower overall by almost half compared with earlier estimates,^2^ and suggest higher incidence in Africa and lower incidence in Asia than previously thought. Whether these differences reflect true changes in typhoid fever epidemiology over time, methodological differences, or both is difficult to know.

Mogasale and colleagues highlight a number of limitations. First, despite the growing number of studies on typhoid fever incidence, the amount of source data remains quite scarce. Furthermore, what constitutes a population-based study of typhoid fever incidence is open to inter pretation. Mogasale and others chose a fairly permissive interpretation to optimise the breadth of data. One consequence is the inclusion of a heterogeneous group of study types that are likely to vary considerably in the completeness of capture of cases. This can be problematic when seeking to understand typhoid fever incidence by age group, when differences in detection by age could have substantial effects on apparent age distribution. Indeed, the age distribution of cases derived from Mogasale and colleagues’ review differs from that measured by very intensive active surveillance in a high incidence setting.^8^

Second, although it is an important and biologically plausible refinement, risk-factor adjustment based on lack of access to improved water in rural areas and urban slums could be open to criticism, as the authors acknowledge. The imperfect relation between access to improved water and consumption of microbiologically safe water is underscored by the occurrence of massive typhoid fever outbreaks in settings with water sources that would be classified as improved.^9^

Third, reliable estimates of typhoid fever complications and death remain elusive. Hospital-based studies can be biased towards severe disease, yet the early detection and treatment of cases inherent and appropriate in high-quality populated-based disease surveillance systems undoubtedly modifies patients’ outcomes.^10,11^ Finally, it is important to ask how the results stack up against other sources of data. Few would question that typhoid fever has declined in a number of Asian countries.^12^ Furthermore, there have been increasing reports of high levels of endemic^13,14^ and epidemic^15,16^ typhoid fever from some locations in Africa. However, studies of community-acquired bloodstream infections suggest that non-typhoidal *Salmonella* has been more common than typhoidal *Salmonella* in sub-Saharan Africa^17^ and national disease surveillance data do not seem consistent with the suggestion that South Africa is a country with a high incidence of typhoid fever.^18^ Indeed, as highlighted by Mogasale and colleagues, incidence estimates for sub-Saharan Africa are heavily influenced by one population-based study from an urban slum in Nairobi, Kenya.^13^ The recently completed multicountry study of typhoid fever incidence in Africa should go some way to providing more data and addressing these concerns.^19^

Burden of disease estimates are foundational to building the investment case for both vaccine and non-vaccine interventions for typhoid fever. Decisions about who would most benefit from vaccination and at what age rely on a clear epidemiological picture. Our picture of typhoid fever burden remains clouded, but Mogasale and colleagues have made refinements that challenge us to think more deeply and to value new data. Soon two new estimates of global typhoid and paratyphoid fever burden, from IHME GBD 2013^20^ and the WHO Foodborne Diseases Burden Epidemiology Reference Group,^21^ will become available. The iterative process of refining and updating burden estimates for typhoid fever is now occurring both consecutively and in parallel, with multiple groups working somewhat independently. Looking to the future, it might be time to take stock of existing estimates and methods, drawing from the strengths of each approach, and striving for both methods that are transparent and results that are timely. Typhoid control would benefit from collective effort to ensure the best possible data to support policy decisions and from a clear message to the world on the scale of the problem.
